# Crystal structure of (*Z*)-3-{3-(4-chloro­phen­yl)-2-[(4-chloro­phen­yl)imino]-2,3-di­hydro­thia­zol-4-yl}-2*H*-chromen-2-one

**DOI:** 10.1107/S1600536814024775

**Published:** 2014-11-19

**Authors:** M. Kayalvizhi, G. Vasuki, R. Raj Kumar, V. Rajeswar Rao

**Affiliations:** a Department of Physics, Kunthavai Naachiar Government Arts College (W) (Autonomous), Thanjavur 613 007, Tamilnadu, India; b Department of Chemistry, National Institute of Technology, Warangal 506 004, Telangana, India

**Keywords:** crystal structure, 2*H*-chromen-2-one, bioactivity, hydrogen bonding, π–π inter­actions

## Abstract

In the title compound, C_24_H_14_Cl_2_N_2_O_2_S, the 2*H*-chromene ring system is approximately planar, with a maximum deviation of 0.025 (2) Å. The thia­zole ring is almost planar, with an r.m.s. deviation of 0.0022 Å, and makes a dihedral angle of 58.52 (7)° with the chromene ring system. The chromene ring system is inclined at angles of 58.3 (1) and 55.39 (9)° with respect to the two chloro­phenyl rings. The two chloro­phenyl rings show significant deviation from coplanarity, with a dihedral angle between them of 47.69 (8)°. The crystal structure features C—H⋯Cl inter­actions extending in (100) and propagating along the *a*-axis direction and weak π–π inter­actions [centroid–centroid separation = 3.867 (2) Å].

## Related literature   

For the bioactivity of coumarin, see: Yusufzai *et al.* (2012 ▶). For related structures, see: Arshad, Osman, Chan *et al.* (2010 ▶); Arshad, Osman, Lam *et al.* (2010*a* ▶,*b* ▶). For synthetic chemistry, medicinal chemistry, photochemistry and solid-state chemistry applications of coumarin derivatives, see: Chopra *et al.* (2009 ▶). For the synthesis, see: Raj Kumar & Rajeswar Rao (2014 ▶).
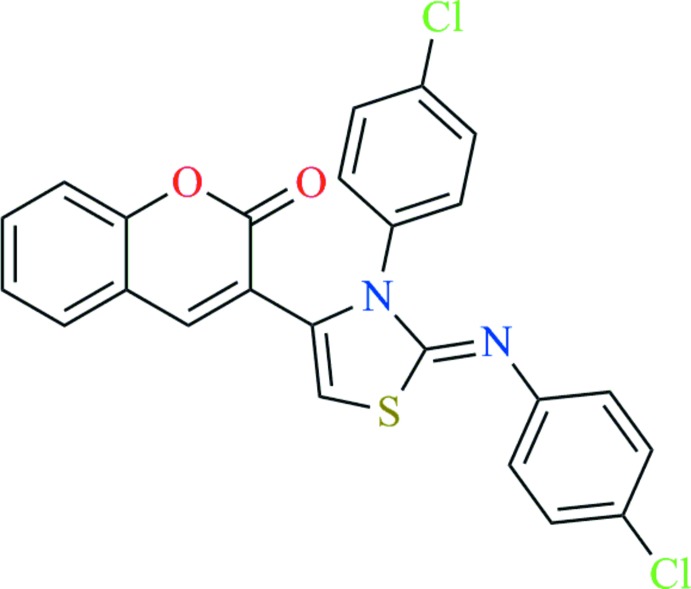


## Experimental   

### Crystal data  

C_24_H_14_Cl_2_N_2_O_2_S
*M* *_r_* = 465.33
Monoclinic, 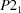

*a* = 9.1491 (7) Å
*b* = 10.3099 (8) Å
*c* = 11.9347 (10) Å
β = 111.587 (2)°
*V* = 1046.80 (14) Å^3^
*Z* = 2
Mo *K*α radiation
μ = 0.44 mm^−1^
*T* = 296 K
0.35 × 0.30 × 0.25 mm

### Data collection  

Bruker Kappa APEXII CCD diffractometer
Absorption correction: multi-scan (*SADABS*; Bruker, 1999 ▶) *T*_min_ = 0.897, *T*_max_ = 1.000
15409 measured reflections
5549 independent reflections
4070 reflections with *I* > 2σ(*I*)
*R*_int_ = 0.024

### Refinement  

*R*[*F* ^2^ > 2σ(*F* ^2^)] = 0.034
*wR*(*F* ^2^) = 0.075
*S* = 1.03
5549 reflections
280 parameters
1 restraint
H-atom parameters constrained
Δρ_max_ = 0.17 e Å^−3^
Δρ_min_ = −0.20 e Å^−3^
Absolute structure: Flack *x* determined using 1604 quotients [(*I* ^+^)−(*I* ^−^)]/[(*I* ^+^)+(*I* ^−^)] (Parsons *et al.*, 2013 ▶)
Absolute structure parameter: −0.004 (19)

### 

Data collection: *APEX2* (Bruker, 2004 ▶); cell refinement: *APEX2* and *SAINT* (Bruker, 2004 ▶); data reduction: *SAINT* and *XPREP* (Bruker, 2004 ▶); program(s) used to solve structure: *SHELXS97* (Sheldrick, 2008 ▶); program(s) used to refine structure: *SHELXL97* (Sheldrick, 2008 ▶); molecular graphics: *ORTEP-3 for Windows* (Farrugia, 2012 ▶); software used to prepare material for publication: *PLATON* (Spek, 2009 ▶).

## Supplementary Material

Crystal structure: contains datablock(s) I, global, 206R. DOI: 10.1107/S1600536814024775/zs2320sup1.cif

Structure factors: contains datablock(s) I. DOI: 10.1107/S1600536814024775/zs2320Isup2.hkl

Supporting information file. DOI: 10.1107/S1600536814024775/zs2320Isup3.cdx

Supporting information file. DOI: 10.1107/S1600536814024775/zs2320Isup4.cml

**Fig. 1.**
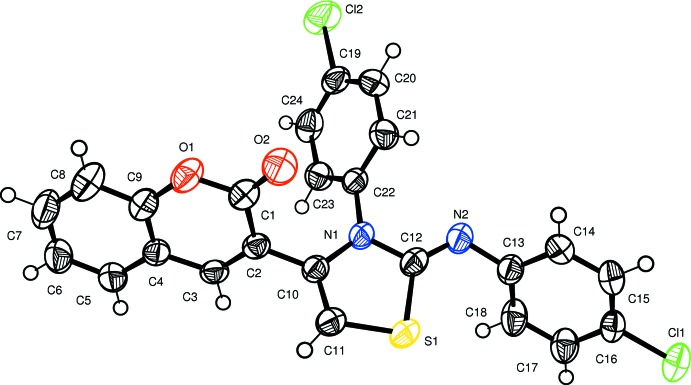
The molecular structure of the title compound showing atom numbering, with displacement ellipsoids drawn at the 50% probability level.

**Fig. 2.**
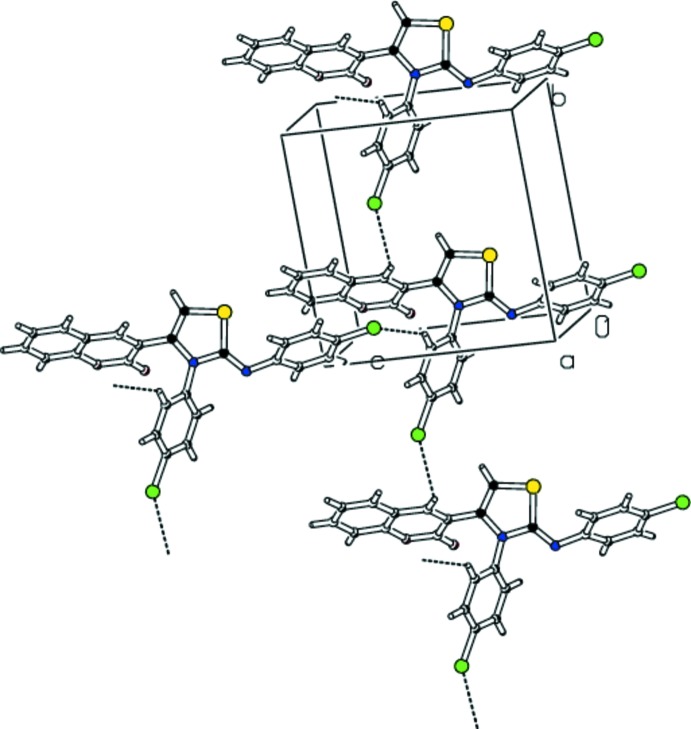
Crystal packing of the title compound in the unit cell, viewed along the a axis, showing C—H···Cl interactions as dashed lines.

. DOI: 10.1107/S1600536814024775/zs2320fig1.tif
The mol­ecular structure of the title compound showing atom numbering, with displacement ellipsoids drawn at the 50% probability level.

a . DOI: 10.1107/S1600536814024775/zs2320fig2.tif
Crystal packing of the title compound in the unit cell, viewed along the *a* axis, showing C—H⋯Cl inter­actions as dashed lines.

**Fig. 3.**
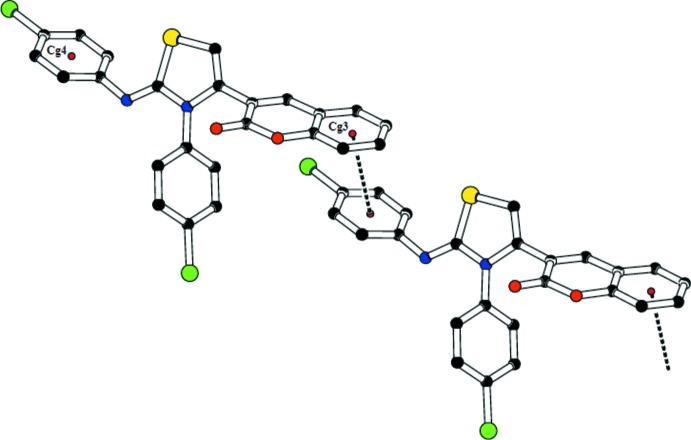
The partial packing of the title compound, showing the π–π interactions.

. DOI: 10.1107/S1600536814024775/zs2320fig3.tif
The partial packing of the title compound, showing the π–π inter­actions.

CCDC reference: 1027667

Additional supporting information:  crystallographic information; 3D view; checkCIF report

## supplementary crystallographic information

### S1. Comment

Compounds containing the coumarin moiety exhibit useful and diverse biological activities (Yusufzai *et al.*, 2012). Coumarins are an important class of organic compounds and have been extensively studied. Such molecules of vast structural diversity find useful applications in several areas of synthetic chemistry, medicinal chemistry and photochemistry. The formation of [2 + 2] cycloaddition products upon irradiation of coumarin and its derivatives has contributed immensely to the area of solid-state chemistry. Several substituted coumarin derivatives find applications in the dye industry and in the area of laser dyes based on the fact that such compounds show state dependent variations in their static dipole moments. The geometry and molecular packing patterns of several coumarins derivatives have been studied to evaluate the features of non-covalent interactions (Chopra *et al.*, 2009). Some of the coumarin derivatives have been found to be useful in photochemotherapy, antitumour, anti-HIV therapy, anti-bacterial, anticoagulant, anti-fungal, cytotoxic activities, free radical scavengers and enzyme inhibiting agents. The related compounds whose structures have been solved by X-ray are 3-{2-[2-(diphenylmethylene)hydrazinyl]thiazol-4-yl}-2*H*-chromen-2-one (Arshad, Osman, Chan *et al.*, 2010), (*Z*)-3-(2-{2-[1-(4-hydroxyphenyl)ethylidene]hydrazin-1-yl}-1,3-thiazol-4-yl)-2*H*-chromen-2-one (Arshad, Osman, Lam *et al.*, 2010*a*) and 3-{2-[2-(2-fluororbenzylidene)hydrazinyl]-1,3-thiazol-4-yl}-2*H*-chromen-2-one (Arshad, Osman, Lam *et al.*, 2010*b*). The title compound, C_24_H_14_Cl_2_N_2_O_2_S, (Fig. 1), is a new derivative of dihydrothiazoyl coumarin. We present herein its crystal structure.

The 2*H*-chromene (O1/C1–C9/O2) ring system is approximately planar, with the maximum deviation of -0.025 (2) Å at atom O1. The thiazole ring (S1/N1/C10–C12) is almost planar with a r.m.s. deviation of 0.0022 Å and makes a dihedral angle of 58.52 (7)° with the chromene ring. The chromene ring system is inclined at angles of 58.3 (1)° and 55.39 (9)° with respect to the two chlorophenyl rings (C13–C18/Cl1) and (C19–C24/Cl2), respectively. The two chlorophenyl rings show significant deviation from coplanarity, with a dihedral angle between the two planes of 47.69 (8)°. The sum of bond angles around N1 [359.79 (5)°] indicates that atom N exhibits *sp*^2^ hybridization. Torsion angles C1—C2—C10—N1 = -58.5 (4)° and C10—N1—C22—C23 = -51.8 (4)° indicate that the chromene ring and the chlorophenyl ring are substituted *synclinally* to the thiazole ring at atoms C2 and C22, respectively. The torsion angle C22—N1—C12—N2 [6.4 (4)°] indicates that the two chlorophenyl rings have a *Z*-configuration across the N1—C12 bond. In the crystal, a short intermolecular C3—H···Cl^i^ contact is observed [3.282 (3) Å] [symmetry code: (i) *x*, *y* - 1, *z*] together with second longer C23—H···Cl1^ii^ contact is observed between C23 and Cl1^ii^ [3.547 (3) Å] [symmetry code: *x* + 1, *y*, *z* + 1] (Fig. 2). Inter-ring π—π stacking interactions between the symmetry related C4–C9 ring (centroid *Cg*3) and the C13–C18^iii^ ring (centroid *Cg*4), with *Cg*3···*Cg*4 = 3.867 (2) Å (symmetry code: (iii) *x* + 1, *y*, *z* + 1) further stabilize the crystal structure (Fig. 3).

### S2. Experimental

The compound was synthesized according to the published procedure (Raj Kumar & Rajeswar Rao, 2014).

### S3. Refinement

All the H atoms were positioned geometrically and treated as riding on their parent atoms: C—H = 0.93 Å, with *U*_iso_(H) = 1.2*U*_eq_(C). Although of no relevance in this achiral stucture, the Flack factor obtained (Parsons *et al.*, 2013) was -0.004 (19).

### Crystal data

**Table:**

C_24_H_14_Cl_2_N_2_O_2_S	*F*(000) = 476
*M**_r_* = 465.33	*D*_x_ = 1.476 Mg m^−^^3^
Monoclinic, *P*2_1_	Mo *K*α radiation, λ = 0.71073 Å
*a* = 9.1491 (7) Å	Cell parameters from 5853 reflections
*b* = 10.3099 (8) Å	θ = 4.8–29.9°
*c* = 11.9347 (10) Å	µ = 0.44 mm^−^^1^
β = 111.587 (2)°	*T* = 296 K
*V* = 1046.80 (14) Å^3^	Block, colourless
*Z* = 2	0.35 × 0.30 × 0.25 mm

### Data collection

**Table:**

Bruker Kappa APEXII CCD diffractometer	5549 independent reflections
Radiation source: fine-focus sealed tube	4070 reflections with *I* > 2σ(*I*)
Graphite monochromator	*R*_int_ = 0.024
ω and φ scans	θ_max_ = 30.2°, θ_min_ = 2.4°
Absorption correction: multi-scan (*SADABS*; Bruker, 1999)	*h* = −12→12
*T*_min_ = 0.897, *T*_max_ = 1.000	*k* = −13→14
15409 measured reflections	*l* = −16→16

### Refinement

**Table:**

Refinement on *F*^2^	Hydrogen site location: inferred from neighbouring sites
Least-squares matrix: full	H-atom parameters constrained
*R*[*F*^2^ > 2σ(*F*^2^)] = 0.034	*w* = 1/[σ^2^(*F*_o_^2^) + (0.0251*P*)^2^ + 0.1767*P*] where *P* = (*F*_o_^2^ + 2*F*_c_^2^)/3
*wR*(*F*^2^) = 0.075	(Δ/σ)_max_ < 0.001
*S* = 1.03	Δρ_max_ = 0.17 e Å^−^^3^
5549 reflections	Δρ_min_ = −0.20 e Å^−^^3^
280 parameters	Absolute structure: Flack *x* determined using 1604 quotients [(*I*^+^)-(*I*^-^)]/[(*I*^+^)+(*I*^-^)] (Parsons *et al.*, 2013)
1 restraint	Absolute structure parameter: −0.004 (19)

### Special details

**Table:**

Geometry. All e.s.d.'s (except the e.s.d. in the dihedral angle between two l.s. planes) are estimated using the full covariance matrix. The cell e.s.d.'s are taken into account individually in the estimation of e.s.d.'s in distances, angles and torsion angles; correlations between e.s.d.'s in cell parameters are only used when they are defined by crystal symmetry. An approximate (isotropic) treatment of cell e.s.d.'s is used for estimating e.s.d.'s involving l.s. planes.

### Fractional atomic coordinates and isotropic or equivalent isotropic displacement parameters (Å^2^)

**Table:**

	*x*	*y*	*z*	*U*_iso_*/*U*_eq_	
Cl1	0.73427 (9)	0.67172 (10)	1.28483 (6)	0.0611 (2)	
Cl2	0.07802 (15)	0.15926 (9)	0.32829 (9)	0.0911 (3)	
S1	0.42551 (10)	0.84490 (8)	0.71595 (7)	0.0526 (2)	
O1	0.2030 (2)	0.7096 (2)	0.17243 (18)	0.0552 (6)	
O2	0.3924 (2)	0.6405 (2)	0.33588 (19)	0.0611 (6)	
N1	0.3118 (2)	0.6598 (2)	0.56482 (18)	0.0408 (5)	
N2	0.4358 (3)	0.5837 (2)	0.7613 (2)	0.0536 (7)	
C1	0.2735 (3)	0.7030 (3)	0.2954 (2)	0.0438 (7)	
C2	0.1989 (3)	0.7740 (3)	0.3656 (2)	0.0366 (6)	
C3	0.0669 (3)	0.8415 (3)	0.3101 (2)	0.0386 (6)	
H3	0.0194	0.8846	0.3562	0.046*	
C4	−0.0030 (3)	0.8489 (3)	0.1811 (2)	0.0392 (6)	
C5	−0.1396 (4)	0.9181 (3)	0.1185 (3)	0.0511 (8)	
H5	−0.1912	0.9631	0.1607	0.061*	
C6	−0.1983 (4)	0.9202 (4)	−0.0050 (3)	0.0606 (9)	
H6	−0.2893	0.9667	−0.0465	0.073*	
C7	−0.1228 (4)	0.8536 (4)	−0.0675 (3)	0.0690 (10)	
H7	−0.1631	0.8562	−0.1513	0.083*	
C8	0.0110 (4)	0.7834 (4)	−0.0087 (3)	0.0645 (9)	
H8	0.0611	0.7378	−0.0515	0.077*	
C9	0.0692 (3)	0.7822 (3)	0.1157 (2)	0.0452 (7)	
C10	0.2782 (3)	0.7744 (3)	0.4978 (2)	0.0379 (6)	
C11	0.3305 (4)	0.8795 (3)	0.5646 (3)	0.0462 (7)	
H11	0.3174	0.9632	0.5333	0.055*	
C12	0.3931 (3)	0.6762 (3)	0.6869 (2)	0.0428 (6)	
C13	0.5081 (3)	0.6096 (3)	0.8852 (3)	0.0453 (7)	
C14	0.6503 (4)	0.5525 (3)	0.9501 (3)	0.0551 (8)	
H14	0.6992	0.5005	0.9106	0.066*	
C15	0.7211 (4)	0.5712 (3)	1.0723 (3)	0.0559 (9)	
H15	0.8178	0.5330	1.1149	0.067*	
C16	0.6480 (3)	0.6468 (3)	1.1310 (2)	0.0422 (6)	
C17	0.5071 (3)	0.7039 (3)	1.0692 (3)	0.0531 (8)	
H17	0.4584	0.7554	1.1092	0.064*	
C18	0.4369 (3)	0.6845 (4)	0.9460 (3)	0.0556 (8)	
H18	0.3402	0.7227	0.9036	0.067*	
C19	0.1469 (4)	0.3047 (3)	0.4032 (3)	0.0506 (8)	
C20	0.3036 (4)	0.3174 (3)	0.4712 (3)	0.0536 (8)	
H20	0.3723	0.2485	0.4792	0.064*	
C21	0.3576 (4)	0.4336 (3)	0.5275 (3)	0.0492 (8)	
H21	0.4636	0.4437	0.5745	0.059*	
C22	0.2553 (3)	0.5348 (3)	0.5145 (2)	0.0377 (6)	
C23	0.0977 (3)	0.5194 (3)	0.4499 (3)	0.0443 (7)	
H23	0.0285	0.5871	0.4448	0.053*	
C24	0.0419 (4)	0.4032 (3)	0.3925 (3)	0.0527 (8)	
H24	−0.0645	0.3920	0.3476	0.063*	

### Atomic displacement parameters (Å^2^)

**Table:**

	*U*^11^	*U*^22^	*U*^33^	*U*^12^	*U*^13^	*U*^23^
Cl1	0.0589 (4)	0.0805 (6)	0.0361 (4)	−0.0094 (4)	0.0086 (3)	0.0030 (4)
Cl2	0.1509 (9)	0.0438 (4)	0.0690 (6)	−0.0333 (6)	0.0292 (6)	−0.0135 (5)
S1	0.0634 (5)	0.0460 (4)	0.0382 (4)	−0.0074 (4)	0.0069 (3)	−0.0095 (3)
O1	0.0514 (11)	0.0756 (15)	0.0401 (11)	0.0026 (11)	0.0187 (10)	−0.0100 (10)
O2	0.0481 (12)	0.0787 (17)	0.0577 (13)	0.0119 (12)	0.0209 (10)	−0.0019 (12)
N1	0.0441 (12)	0.0374 (11)	0.0330 (11)	−0.0043 (11)	0.0050 (9)	−0.0020 (11)
N2	0.0641 (17)	0.0458 (15)	0.0380 (13)	−0.0036 (12)	0.0037 (12)	−0.0006 (11)
C1	0.0427 (15)	0.0492 (16)	0.0404 (15)	−0.0036 (14)	0.0162 (13)	−0.0047 (13)
C2	0.0404 (15)	0.0341 (13)	0.0357 (14)	−0.0053 (12)	0.0144 (12)	−0.0038 (11)
C3	0.0476 (15)	0.0300 (12)	0.0390 (14)	−0.0045 (13)	0.0170 (12)	−0.0054 (12)
C4	0.0445 (14)	0.0337 (13)	0.0367 (13)	−0.0076 (13)	0.0117 (12)	−0.0020 (12)
C5	0.0561 (19)	0.0434 (16)	0.0460 (17)	0.0003 (14)	0.0096 (15)	0.0001 (14)
C6	0.063 (2)	0.058 (2)	0.0460 (18)	0.0005 (17)	0.0026 (16)	0.0053 (16)
C7	0.078 (2)	0.082 (2)	0.0355 (16)	−0.005 (2)	0.0079 (17)	0.0021 (19)
C8	0.066 (2)	0.088 (3)	0.0402 (17)	−0.007 (2)	0.0208 (16)	−0.0091 (18)
C9	0.0461 (16)	0.0518 (17)	0.0372 (15)	−0.0077 (14)	0.0146 (13)	−0.0026 (13)
C10	0.0371 (14)	0.0396 (15)	0.0352 (14)	−0.0007 (12)	0.0112 (12)	−0.0027 (12)
C11	0.0566 (19)	0.0402 (17)	0.0398 (16)	−0.0039 (13)	0.0154 (14)	−0.0043 (12)
C12	0.0399 (13)	0.0471 (17)	0.0370 (14)	−0.0047 (14)	0.0090 (11)	−0.0071 (14)
C13	0.0475 (16)	0.0430 (15)	0.0381 (15)	−0.0052 (13)	0.0069 (13)	0.0019 (13)
C14	0.066 (2)	0.0477 (17)	0.0456 (17)	0.0145 (16)	0.0142 (16)	0.0030 (14)
C15	0.0490 (18)	0.061 (2)	0.0464 (18)	0.0120 (15)	0.0039 (15)	0.0077 (15)
C16	0.0402 (14)	0.0489 (16)	0.0332 (13)	−0.0061 (13)	0.0086 (11)	0.0064 (13)
C17	0.0462 (16)	0.072 (2)	0.0430 (16)	0.0079 (16)	0.0182 (13)	0.0052 (15)
C18	0.0384 (14)	0.081 (2)	0.0429 (16)	0.0096 (17)	0.0095 (13)	0.0070 (18)
C19	0.079 (2)	0.0358 (15)	0.0362 (15)	−0.0115 (15)	0.0207 (15)	−0.0025 (12)
C20	0.072 (2)	0.0378 (17)	0.0506 (18)	0.0081 (14)	0.0218 (17)	0.0017 (13)
C21	0.0434 (17)	0.0474 (18)	0.0496 (18)	0.0029 (14)	0.0089 (14)	−0.0011 (14)
C22	0.0441 (16)	0.0356 (14)	0.0315 (13)	−0.0028 (12)	0.0118 (12)	−0.0021 (11)
C23	0.0390 (15)	0.0445 (17)	0.0459 (16)	−0.0006 (12)	0.0114 (13)	−0.0011 (13)
C24	0.0486 (17)	0.0565 (19)	0.0447 (17)	−0.0186 (16)	0.0075 (14)	0.0003 (15)

### Geometric parameters (Å, º)

**Table:**

Cl1—C16	1.731 (3)	C8—C9	1.380 (4)
Cl2—C19	1.741 (3)	C8—H8	0.9300
S1—C11	1.729 (3)	C10—C11	1.326 (4)
S1—C12	1.777 (3)	C11—H11	0.9300
O1—C1	1.371 (3)	C13—C18	1.376 (4)
O1—C9	1.382 (4)	C13—C14	1.378 (4)
O2—C1	1.203 (3)	C14—C15	1.374 (4)
N1—C12	1.381 (3)	C14—H14	0.9300
N1—C10	1.396 (4)	C15—C16	1.375 (4)
N1—C22	1.436 (3)	C15—H15	0.9300
N2—C12	1.263 (4)	C16—C17	1.362 (4)
N2—C13	1.406 (4)	C17—C18	1.386 (4)
C1—C2	1.457 (4)	C17—H17	0.9300
C2—C3	1.339 (4)	C18—H18	0.9300
C2—C10	1.474 (4)	C19—C20	1.369 (5)
C3—C4	1.435 (3)	C19—C24	1.371 (5)
C3—H3	0.9300	C20—C21	1.373 (4)
C4—C9	1.378 (4)	C20—H20	0.9300
C4—C5	1.394 (4)	C21—C22	1.371 (4)
C5—C6	1.371 (4)	C21—H21	0.9300
C5—H5	0.9300	C22—C23	1.371 (4)
C6—C7	1.372 (5)	C23—C24	1.381 (4)
C6—H6	0.9300	C23—H23	0.9300
C7—C8	1.372 (5)	C24—H24	0.9300
C7—H7	0.9300		
			
C11—S1—C12	90.84 (13)	N2—C12—N1	123.8 (3)
C1—O1—C9	122.3 (2)	N2—C12—S1	128.0 (2)
C12—N1—C10	114.9 (2)	N1—C12—S1	108.2 (2)
C12—N1—C22	121.5 (2)	C18—C13—C14	118.4 (3)
C10—N1—C22	123.41 (19)	C18—C13—N2	122.1 (3)
C12—N2—C13	120.0 (3)	C14—C13—N2	119.4 (3)
O2—C1—O1	117.1 (3)	C15—C14—C13	121.0 (3)
O2—C1—C2	125.8 (3)	C15—C14—H14	119.5
O1—C1—C2	117.1 (2)	C13—C14—H14	119.5
C3—C2—C1	120.3 (2)	C14—C15—C16	119.5 (3)
C3—C2—C10	121.8 (2)	C14—C15—H15	120.2
C1—C2—C10	117.8 (2)	C16—C15—H15	120.2
C2—C3—C4	121.5 (3)	C17—C16—C15	120.7 (3)
C2—C3—H3	119.2	C17—C16—Cl1	118.8 (2)
C4—C3—H3	119.2	C15—C16—Cl1	120.5 (2)
C9—C4—C5	118.3 (3)	C16—C17—C18	119.2 (3)
C9—C4—C3	117.6 (2)	C16—C17—H17	120.4
C5—C4—C3	124.1 (3)	C18—C17—H17	120.4
C6—C5—C4	120.2 (3)	C13—C18—C17	121.1 (3)
C6—C5—H5	119.9	C13—C18—H18	119.5
C4—C5—H5	119.9	C17—C18—H18	119.5
C5—C6—C7	120.0 (3)	C20—C19—C24	121.9 (3)
C5—C6—H6	120.0	C20—C19—Cl2	119.3 (3)
C7—C6—H6	120.0	C24—C19—Cl2	118.7 (3)
C6—C7—C8	121.2 (3)	C19—C20—C21	118.9 (3)
C6—C7—H7	119.4	C19—C20—H20	120.5
C8—C7—H7	119.4	C21—C20—H20	120.5
C7—C8—C9	118.3 (3)	C22—C21—C20	120.0 (3)
C7—C8—H8	120.9	C22—C21—H21	120.0
C9—C8—H8	120.9	C20—C21—H21	120.0
C4—C9—C8	122.0 (3)	C23—C22—C21	120.6 (3)
C4—C9—O1	121.1 (2)	C23—C22—N1	118.7 (2)
C8—C9—O1	117.0 (3)	C21—C22—N1	120.7 (2)
C11—C10—N1	113.1 (2)	C22—C23—C24	120.0 (3)
C11—C10—C2	124.9 (3)	C22—C23—H23	120.0
N1—C10—C2	121.9 (2)	C24—C23—H23	120.0
C10—C11—S1	113.0 (2)	C19—C24—C23	118.5 (3)
C10—C11—H11	123.5	C19—C24—H24	120.7
S1—C11—H11	123.5	C23—C24—H24	120.7
			
C9—O1—C1—O2	−177.6 (3)	C13—N2—C12—N1	−175.3 (3)
C9—O1—C1—C2	1.5 (4)	C13—N2—C12—S1	5.4 (4)
O2—C1—C2—C3	179.3 (3)	C10—N1—C12—N2	−178.8 (3)
O1—C1—C2—C3	0.3 (4)	C22—N1—C12—N2	6.4 (4)
O2—C1—C2—C10	2.7 (4)	C10—N1—C12—S1	0.5 (3)
O1—C1—C2—C10	−176.4 (2)	C22—N1—C12—S1	−174.3 (2)
C1—C2—C3—C4	−1.5 (4)	C11—S1—C12—N2	178.9 (3)
C10—C2—C3—C4	175.0 (2)	C11—S1—C12—N1	−0.4 (2)
C2—C3—C4—C9	1.0 (4)	C12—N2—C13—C18	56.7 (4)
C2—C3—C4—C5	−179.7 (3)	C12—N2—C13—C14	−127.2 (3)
C9—C4—C5—C6	−0.7 (4)	C18—C13—C14—C15	−1.0 (5)
C3—C4—C5—C6	179.9 (3)	N2—C13—C14—C15	−177.2 (3)
C4—C5—C6—C7	0.2 (5)	C13—C14—C15—C16	0.8 (5)
C5—C6—C7—C8	0.5 (6)	C14—C15—C16—C17	−0.5 (5)
C6—C7—C8—C9	−0.6 (6)	C14—C15—C16—Cl1	179.9 (3)
C5—C4—C9—C8	0.6 (4)	C15—C16—C17—C18	0.4 (5)
C3—C4—C9—C8	−180.0 (3)	Cl1—C16—C17—C18	−180.0 (3)
C5—C4—C9—O1	−178.6 (3)	C14—C13—C18—C17	0.9 (5)
C3—C4—C9—O1	0.8 (4)	N2—C13—C18—C17	177.0 (3)
C7—C8—C9—C4	0.1 (5)	C16—C17—C18—C13	−0.6 (5)
C7—C8—C9—O1	179.3 (3)	C24—C19—C20—C21	−1.9 (5)
C1—O1—C9—C4	−2.0 (4)	Cl2—C19—C20—C21	178.3 (2)
C1—O1—C9—C8	178.7 (3)	C19—C20—C21—C22	−0.4 (5)
C12—N1—C10—C11	−0.4 (3)	C20—C21—C22—C23	3.0 (5)
C22—N1—C10—C11	174.3 (3)	C20—C21—C22—N1	−175.1 (3)
C12—N1—C10—C2	177.1 (2)	C12—N1—C22—C23	122.6 (3)
C22—N1—C10—C2	−8.2 (4)	C10—N1—C22—C23	−51.8 (4)
C3—C2—C10—C11	−58.0 (4)	C12—N1—C22—C21	−59.4 (4)
C1—C2—C10—C11	118.6 (3)	C10—N1—C22—C21	126.3 (3)
C3—C2—C10—N1	124.9 (3)	C21—C22—C23—C24	−3.2 (4)
C1—C2—C10—N1	−58.5 (4)	N1—C22—C23—C24	174.9 (3)
N1—C10—C11—S1	0.0 (3)	C20—C19—C24—C23	1.7 (5)
C2—C10—C11—S1	−177.3 (2)	Cl2—C19—C24—C23	−178.6 (2)
C12—S1—C11—C10	0.2 (3)	C22—C23—C24—C19	0.9 (4)
