# Supplementary figures and images for: Crystal structure of (Z)-3-{3-(4-chloro­phen­yl)-2-[(4-chloro­phen­yl)imino]-2,3-di­hydro­thia­zol-4-yl}-2H-chromen-2-one
Source: Acta Crystallogr Sect E Struct Rep Online. 2014 Nov 19;70(Pt 12):o1268–9. doi: 10.1107/S1600536814024775 (PMC4257461; doi:10.1107/S1600536814024775)

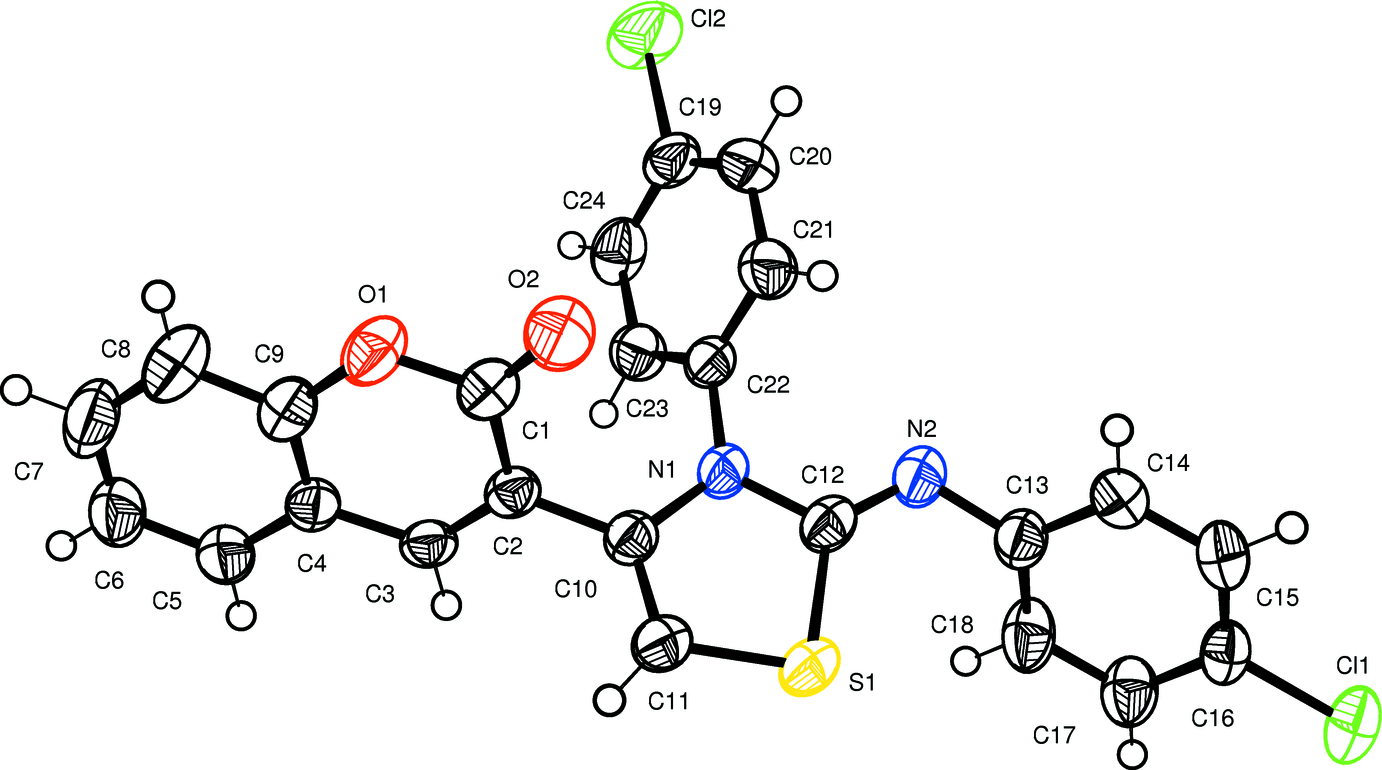

Supplement: Supplementary file 5 [file e-70-o1268-fig1.tif]

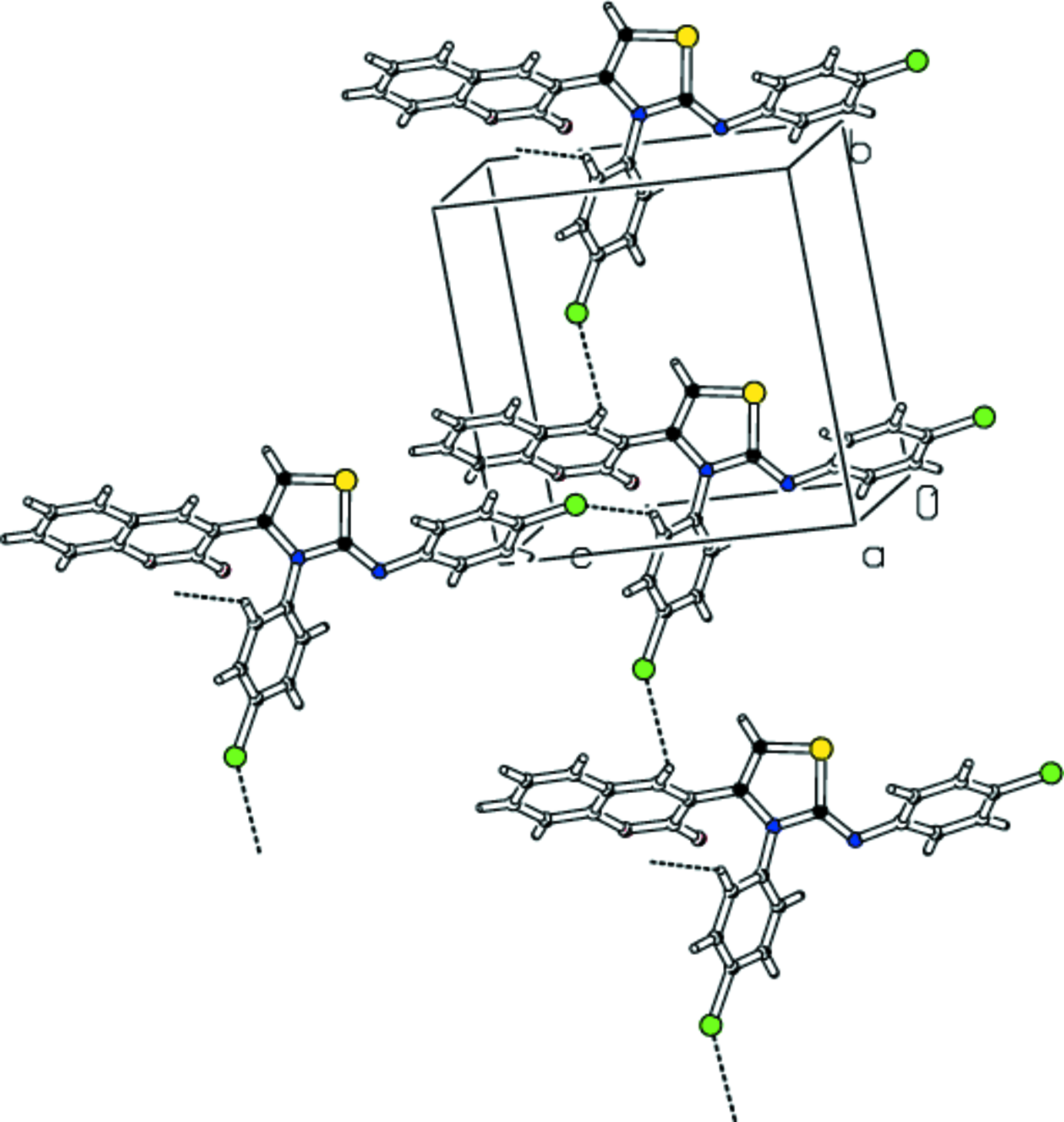

Supplement: Supplementary file 6 [file e-70-o1268-fig2.tif]

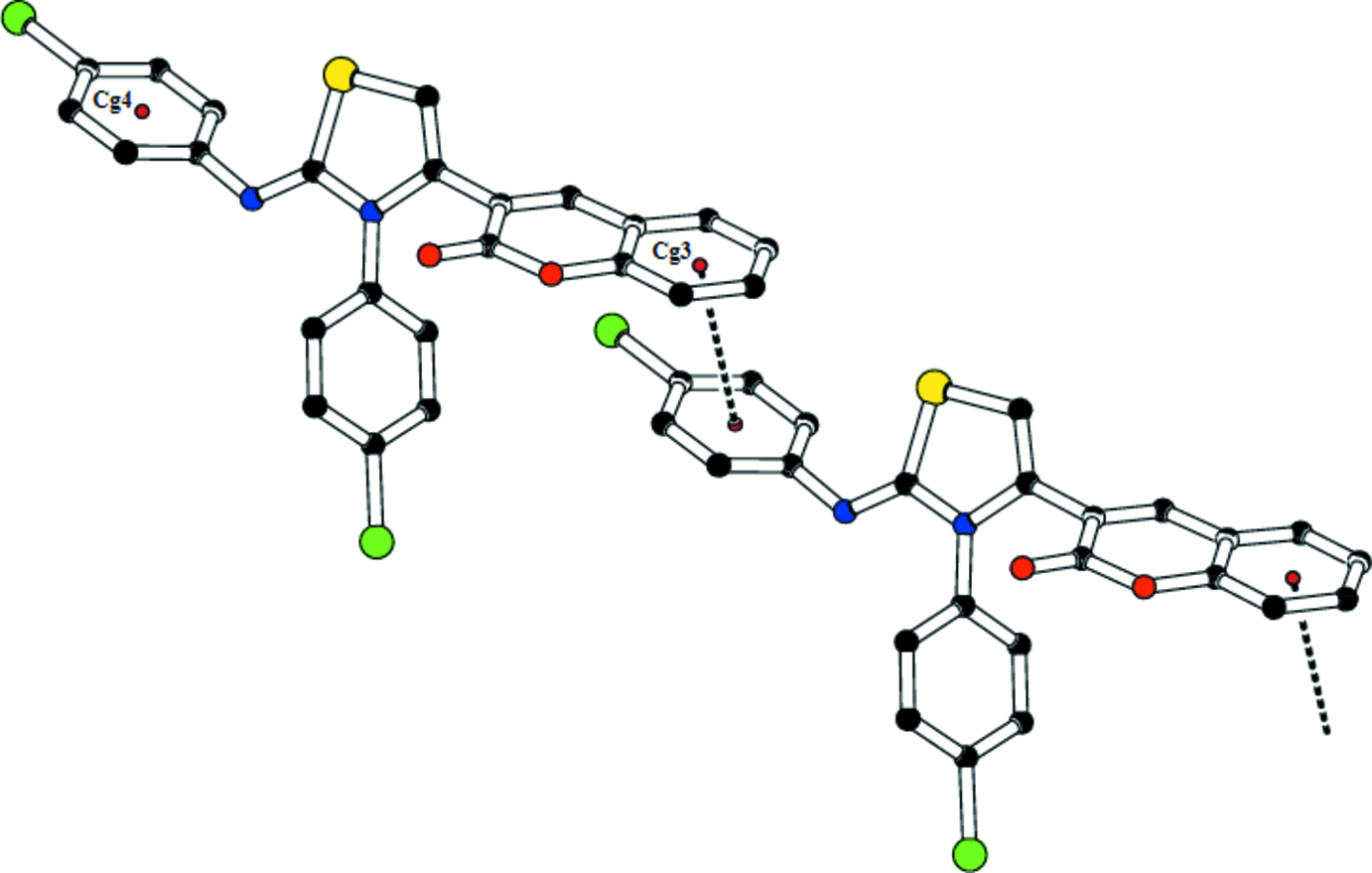

Supplement: Supplementary file 7 [file e-70-o1268-fig3.tif]
